# Autoantibodies in the Extraintestinal Manifestations of Celiac Disease

**DOI:** 10.3390/nu10081123

**Published:** 2018-08-20

**Authors:** Xuechen B. Yu, Melanie Uhde, Peter H. Green, Armin Alaedini

**Affiliations:** 1 Department of Medicine, Columbia University Medical Center, 1130 Saint Nicholas Ave., New York, NY 10032, USA; xy2314@cumc.columbia.edu (X.B.Y.); melanieuhde86@gmail.com (M.U.); pg11@columbia.edu (P.H.G.); 2 Celiac Disease Center, Columbia University, New York, NY 10032, USA; 3 Institute of Human Nutrition, Columbia University, New York, NY 10032, USA

**Keywords:** celiac disease, gluten, gliadin, autoantibody, B cell, T cell, transglutaminase, synapsin, ganglioside, gluten sensitivity, gastrointestinal symptoms, molecular mimicry, intermolecular help, biomarker

## Abstract

Increased antibody reactivity towards self-antigens is often indicative of a disruption of homeostatic immune pathways in the body. In celiac disease, an autoimmune enteropathy triggered by the ingestion of gluten from wheat and related cereals in genetically predisposed individuals, autoantibody reactivity to transglutaminase 2 is reflective of the pathogenic role of the enzyme in driving the associated inflammatory immune response. Autoantibody reactivity to transglutaminase 2 closely corresponds with the gluten intake and clinical presentation in affected patients, serving as a highly useful biomarker in the diagnosis of celiac disease. In addition to gastrointestinal symptoms, celiac disease is associated with a number of extraintestinal manifestations, including those affecting skin, bones, and the nervous system. Investigations of these manifestations in celiac disease have identified a number of associated immune abnormalities, including B cell reactivity towards various autoantigens, such as transglutaminase 3, transglutaminase 6, synapsin I, gangliosides, and collagen. Clinical relevance, pathogenic potential, mechanism of development, and diagnostic and prognostic value of the various identified autoantibody reactivities continue to be subjects of investigation and will be reviewed here.

## 1. Introduction

Celiac disease is a T cell-mediated systemic autoimmune disease triggered by the ingestion of wheat gluten and related proteins in rye and barley in genetically susceptible individuals [1,2,3]. Gluten is comprised of approximately 70 different proteins (including various gliadins and glutenins), which share a number of immunogenic amino acid sequences found to be pathogenic in the context of celiac disease [4]. Celiac disease has a strong genetic component, with approximately 95% of patients expressing the human leukocyte antigens (HLA)-DQ2 and HLA-DQ8 [5]. The frequency of HLA-DQ2 and -DQ8 and the per capita consumption of wheat are important determinants of celiac disease prevalence [6]. Although formerly considered as a rare condition mainly affecting children, celiac disease is now recognized as one of the most common autoimmune disorders and estimated to affect approximately 1% of the population worldwide [7]. In North America and Europe, celiac disease prevalence has been found to be increasing in recent decades [2,8,9]. The frequency of celiac is also rising in Asia-Pacific regions, where traditional rice-based diets are increasingly replaced by Western-style diets with a larger content of wheat-containing products [10].

The increasing prevalence of celiac disease is also partly attributed to the recent development of highly specific and sensitive serologic tests, which allow for safe and effective screening in individuals suspected of having celiac disease [11]. The gold standard for the diagnosis of celiac disease is based on endoscopic biopsy of the small intestine demonstrating the characteristic histologic features of duodenal lymphocyte infiltration, crypt hyperplasia, and villous atrophy, further supported by positive serologic tests, and the confirmation of remission of symptoms upon the removal of gluten-containing foods from diet [11,12]. Complete elimination of gluten-containing food from diet is currently the only effective and safe treatment for celiac disease [13,14].

The classic gastrointestinal symptoms of celiac disease includes diarrhea, abdominal pain, and malabsorption. However, celiac disease is often also associated with a number of extraintestinal manifestations such as osteoporosis, anemia, and dermatitis herpetiformis, among others [3,14,15]. The clinical picture in some patients can also include other autoimmune diseases, including type 1 diabetes, autoimmune thyroiditis, and autoimmune hepatitis, likely due in part to shared genetic factors [5,16,17]. 

The production of autoantibodies is an important feature of many autoimmune diseases, indicating a loss of immune tolerance to self-antigens. In celiac disease, a significantly enhanced autoantibody response to the transglutaminase 2 (TG2) enzyme, also known as tissue transglutaminase (tTG), is a hallmark of the pathogenic process and signifies a loss of tolerance to wheat gluten [11,18]. In addition to antibodies against TG2, other autoantibodies have also been reported in patients with celiac disease, particularly in the context of extraintestinal manifestations. In this review, the origin, development, and the potential pathogenic role of autoantibodies associated with extraintestinal manifestations of celiac disease are discussed. 

## 2. Transglutaminases

### 2.1. Transglutaminase 2

By the 1960s, it was clear that, in addition to antibodies against the immunostimulatory gluten proteins of wheat and related cereals, patients with celiac disease express elevated autoantibody responses to the connective tissue surrounding smooth muscle fibers [19,20]. These antibodies became known as anti-endomysial and anti-reticulin antibodies [21,22,23]. It was not until 1997, when Dieterich et al. identified TG2 as the major endomysial target autoantigen [24]. Later research has shown that the target of the so-called anti-reticulin antibodies was also TG2 [25]. 

TG2 is a member of the structurally and functionally related group of transglutaminase proteins that catalyze the modification of proteins by introducing covalent bonds between amine groups (such as a lysine) and γ-carboxamide groups of peptide-bound glutamines [26]. TG2, the first transglutaminase discovered, is unique in some aspects, including that it is a ubiquitously expressed enzyme expressed in various tissues and cell types, and in various locations inside the cell and at the cell surface [26]. Under certain conditions, TG2 may react with H_2_O in preference over an amine, converting a specific glutamine to glutamate via deamidation [27]. Intracellular TG2 is involved in signaling events that support cell survival in response to wounding, hypoxia, and oxidative stress [27]. Extracellular TG2 has a role in the regulation of the cytoskeleton by crosslinking extracellular matrix proteins such as fibronectin and integrins, and is believed to function in cell adhesion, matrix assembly, and cell motility [28]. 

TG2 plays a central role in the initiation of immune reactivity towards dietary gluten in the context of celiac disease [29,30], which also involves the celiac disease susceptibility genes, HLA-DQ2 and -DQ8 [31]. TG2 can effectively deamidate specific glutamine residues of gluten sequences that may have crossed the epithelial barrier and found access to the lamina propria. Antigen presenting cells expressing the HLA-DQ2 and HLA-DQ8 molecules have an increased affinity for these deamidated peptides [32]. Subsequent binding of the generated immunogenic peptides to the HLA molecules results in peptide complexes that can activate host gluten specific CD4^+^ T cells in the lamina propria. Activation of these T cells is accompanied by the production of a number of cytokines that can in turn promote inflammation and villous damage in the small intestine through the release of metalloproteinases by fibroblasts and inflammatory cells, as well as providing help to activate gluten-specific B cell responses [30,33].

Gluten-specific CD4^+^ T cells are speculated to stimulate B cell production of not only anti-gluten antibody, but also anti-TG2 antibody. In the absence of any TG2-specific T cells being identified, the anti-TG2 antibody response is believed to be driven by a process referred to as intermolecular help, similar to the hapten-carrier system. Accordingly, gluten-specific CD4^+^ T cells are thought to provide help to TG2-specific B cells when TG2–gluten complexes are formed [34]. Such a gluten-specific T cell-driven mechanism would lead to an anti-TG2 immune response without the requirement for TG2-specific T cells. With repeated exposure to TG2–gluten complexes, affinity maturation towards the TG2 antigen can potentially generate specific high-affinity autoantibody reactivity [35,36,37]. Anti-TG2 autoantibodies are thus gluten-dependent, and the antibody titer decreases rapidly after the elimination of gluten from diet [18,38]. IgA anti-TG2 autoantibodies have high specificity (>90%) and sensitivity (>95%) in celiac disease, currently serving as a particularly useful aid in diagnosis [18,39]. 

Whether antibodies to TG2 can play a clear role in disease pathogenesis in humans has not been definitively proven. Anti-TG2 antibodies bind to several epitopes of TG2, including the enzymatic core of the protein, and can thus interfere with TG2 bioactivity [40]. As TG2 is involved in epithelial cell differentiation through activation of transforming growth factor β [41], anti-TG2 autoantibodies have been shown to reduce epithelial cell differentiation, increase epithelial cell permeability in an intestinal cell line, and induce monocyte activation upon binding to Toll-like receptor 4 [42,43,44]. Data from in vitro studies indicate that anti-TG2 antibodies detected along the villous and crypt basement membranes in the jejunum from celiac disease patients may take part in the intestinal damage, particularly the remodeling of the small bowel mucosal architecture and the development of villous atrophy as well as crypt hyperplasia [43,45,46,47]. 

Because TG2 is the most widely expressed member of the transglutaminase family of proteins in the body, being present in almost all cell types, and participates in various biological reactions, the autoantibodies have the potential to negatively affect the activity of the enzyme and its biological role in tissues outside of the gastrointestinal tract as well [48]. The presence of IgA deposits colocalizing with TG2 in the liver, lymph nodes, muscle, thyroid, bone, and brain indicates that the circulating autoantibodies originating from the small intestine can access the autoantigen throughout the body and may potentially exert certain pathogenic effects [42,49,50,51]. Although the significance of anti-TG2 antibody binding to thyroid tissue or bone is not clear, both thyroid dysfunction and reduced bone density are common in celiac disease, raising the possibility that the antibodies may affect target organ function [49,50,52]. Anti-TG2 autoantibodies found within the muscular layer of brain vessels have also been speculated to cause disruption of the blood–brain barrier, which may further expose the central nervous system to other autoantibodies and potential toxins [51]. In mice, the injection of anti-TG2 antibodies in the lateral ventricle of the brain has been shown to cause deficits in motor coordination [53]. The data suggest that once exposed to the central nervous system, anti-TG2 antibodies may play a role in inducing neurologic deficits. Anti-TG2 antibodies isolated from celiac disease patients also have the potential to cross-react with other members of the transglutaminase family of enzymes due to some level of sequence homology, suggesting that such autoantibodies may additionally affect the activity of other transglutaminases [53]. Taken together, the data are suggestive of a pathogenic role for anti-TG2 autoantibodies in some of the extraintestinal manifestations of celiac disease.

### 2.2. Transglutaminase 3

The skin manifestation of celiac disease, known as dermatitis herpetiformis, was first described by Louis Adolphus Duhring in 1884 as an itchy, blistering, skin disease [54]. Dermatitis herpetiformis is characterized by the deposition of pathognomonic granular IgA in the dermal papillae, sometimes without significant gastrointestinal symptoms, and shares the same HLA associations with celiac disease [5]. In 2002, epidermal transglutaminase (eTG), also known as transglutaminase 3 (TG3), was identified as the main autoantigen target in skin IgA deposits in dermatitis herpetiformis [55]. In addition to increased anti-TG2 antibodies, dermatitis herpetiformis patients also present with elevated levels of antibody directed at TG3 [55,56].

TG3 is mainly expressed in the cornified layer of the epidermis and has been shown to play an important role in epidermal keratinization and in the formation of cornified envelope, which is essential for the maintenance for skin homeostasis [57]. Serum levels of anti-TG2 and anti-TG3 antibodies appear to correlate in celiac disease patients without skin manifestation, but not in patients with dermatitis herpetiformis, suggesting there is antibody reactivity to specific non-cross-reactive epitopes of TG3 in dermatitis herpetiformis [55,58]. IgA autoantibodies against TG3 have been reported to be detected in as much as 95% of dermatitis herpetiformis patients, substantially more than those against TG2 (79% of patients) [59]. In addition, detection of IgA antibody to TG3 has been found to efficiently distinguish untreated dermatitis herpetiformis from other dermatological itchy diseases and to be highly sensitive to a gluten-free diet [60]. IgA anti-TG3 antibody has been proposed as a useful diagnostic marker for dermatitis herpetiformis in both pediatric and adult patients [59,60,61].

The production of anti-TG3 antibodies may begin as a result of cross-reactivity of anti-TG2 IgA antibodies with TG3, which is released from epidermal keratinocytes and can diffuse through the basement membrane in regions of trauma [62]. Prolonged gluten immune stimulation may allow for epitope spreading and further maturation of these antibodies, resulting in the development of high affinity anti-TG3 antibodies [55,62,63]. Disappearance of anti-TG3 IgA antibody in response to dietary exclusion of gluten is slow and may take longer than for antibody response to TG2, suggesting that mechanisms other than homology between TG2 and TG3 might trigger the production of anti-TG3 antibodies [64,65,66]. 

Deposition of IgA antibodies in dermatitis herpetiformis is believed to play a role in the infiltration of neutrophils into the papillary dermis and in the formation of basement membrane zone vesicles in the lamina lucida [55]. TG3 in the papillary dermis has been found to overlap with the deposits of IgA antibodies in dermatitis herpetiformis patients, implying that TG3 is bound by the IgA autoantibodies [55,63]. It is hypothesized that active TG3 may cross-link anti-TG3 antibodies to certain dermal structural elements, leading to the dermal deposition of anti-TG3 IgA, which can in turn invoke skin pathology such as the associated blisters and papules [55]. However, anti-TG3 IgA deposits have also been found in uninvolved skin in affected patients, in areas away from lesions, suggesting that factors beyond these immune complexes may be necessary for lesion formation [62,63]. 

### 2.3. Transglutaminase 6

In addition to the well-characterized gastrointestinal and skin manifestations, a number of studies have reported on various other symptoms associated with celiac disease. Neurologic deficits, including peripheral neuropathy and cerebellar ataxia, are among the most common extraintestinal symptoms reported in conjunction with celiac disease [67,68]. Furthermore, elevated levels of anti-gliadin antibody have been associated with idiopathic neuropathy and idiopathic ataxia, even in the apparent absence of the characteristic mucosal pathology [69,70,71,72]. The terms “gluten ataxia” and “gluten neuropathy” have been used to describe these conditions, although the significance of the anti-gliadin antibodies in the absence of biopsy-proven intestinal damage has been debated [70,72,73]. 

Among these, idiopathic or sporadic ataxia associated with anti-gliadin antibodies has been the best studied in terms of understanding its frequency in different populations of ataxia patients and its potential etiology and pathogenic mechanism. A recent meta-analysis of several studies further validates the presence of significantly increased levels of antibody to gliadin among patients with non-hereditary ataxia [74]. There have been suggestions that gluten ataxia would fit better within the spectrum of non-celiac wheat/gluten sensitivity (NCWS) rather than celiac disease, based on serologic, histologic, and genetic markers [75,76]. In 2008, a novel neuronal transglutaminase, TG6, was reported as a target autoantigen in patients with gluten ataxia [77]. Antibodies to TG6 were later also detected in patients with gluten neuropathy [78]. However, the specificity of anti-TG6 antibodies in gluten ataxia and gluten neuropathy needs further investigation, as other studies have found such antibodies in patients with other conditions and in those without neurologic symptoms as well [77,78,79,80,81].

TG6 is predominantly expressed in a subset of neurons and plays a role in neurogenesis, particularly in the context of nervous system development and motor function [82]. TG6 is encoded on the same chromosome (20q11–12) as TG2 in humans [83]. Similarly to TG2, when TG6 is incubated with gluten peptides, it can both deamidate and transamidate glutamine residues, and there is a large degree of overlap in glutamine donor substrates of TG6 and TG2 [82]. TG6 can also form the previously mentioned hapten-carrier complexes with gluten, but to a lesser extent when compared with TG2 [84]. Therefore, it is conceivable that, in the event of blood–brain or blood–nerve barrier disruption, TG6 may become exposed to gluten-derived antigens. As such, gluten-specific CD4^+^ T cells may be able to provide help to TG6-specific B cells, leading to the production of anti-TG6 autoantibodies in a similar fashion to anti-TG2 antibodies. 

The relationship between gluten intake and the development of anti-TG6 antibodies has been examined. Lindfors et al. did not observe a decrease in anti-TG6 IgA antibodies in celiac disease patients on a gluten-free diet, suggesting a lack of gluten-dependency of TG6 autoantibodies [81]. However, a more recent study on a pediatric cohort of celiac disease patients found anti-TG6 antibody reactivity to correlate with the duration of gluten exposure, and to decline in response to the introduction of a gluten-free diet [79].

Similar to what has been described for anti-TG2 autoantibodies, anti-TG6 antibodies have the potential to disrupt TG6’s biological functions. In mice, the injection of celiac disease patient-derived TG2 antibody that can cross-react with TG6 into mouse brain has been shown to cause deficits in motor coordination [53]. Recently, missense mutations in the of transglutaminase 6 gene have been identified in families of Chinese patients with spinocerebellar ataxia type 35 (SCA35) [85]. However, a causative link between neurological manifestations and autoantibodies to TG6 remains unclear [86]. Future studies on anti-TG6 antibodies can help in further clarifying their diagnostic and pathogenic potential in the context of neurologic and other manifestations in celiac disease and gluten sensitivity. 

## 3. Gangliosides

Gangliosides are sialic acid-containing glycosphingolipids present in high concentrations in the nervous system, as well as on gut epithelial cells [87,88]. Antibodies to gangliosides, especially GM1, GD1a, GD1b, and GQ1b, are associated with and serve as diagnostic markers for a number of immune-mediated peripheral neuropathies, such as multifocal motor neuropathy and Guillain-Barré syndrome [89,90,91]. The antibodies are directed at carbohydrate epitopes of the ganglioside molecule [89]. The presence of anti-ganglioside antibodies in celiac disease patients with peripheral neuropathy was first reported by our team in 2002 [92]. A number of subsequent studies have confirmed the presence of various anti-ganglioside antibodies in conjunction with neurologic symptoms in celiac disease patients and those with immune reactivity to gluten [93,94,95,96,97,98,99]. At least one study has found that anti-ganglioside antibody reactivity responds to the exclusion of gluten from diet in a significant subset of patients with celiac disease [96]. 

Generation of anti-ganglioside antibodies is speculated to be linked to the intestinal immune response to ingested gluten. In acute immune-mediated neuropathies, the presence of anti-ganglioside antibodies has been demonstrated to result from molecular mimicry between gangliosides and bacterial or viral oligosaccharides [100,101]. While some gliadins may be glycosylated, epitopes that resemble gangliosides have not been found [102], making molecular mimicry less likely. However, it should be noted that while most gluten proteins appear to bear few or no carbohydrates, glycosylation of gluten can take place during or after the processing of flour and especially in food preparation under elevated temperatures [103]. A role for such Maillard reaction modifications of gluten in triggering an immune response that may target other autoantigens cannot be ruled out.

An alternative mechanism by which the antibody response to GM1 and other gangliosides could be generated may be through intermolecular help in a way similar to anti-TG2 autoantibodies in celiac disease [30]. In fact, it has been shown that gliadin can bind to the ganglioside-rich intestinal brush border membrane in an enzyme-independent way and can form stable complexes with GM1 ganglioside that are resistant to denaturing conditions [102]. The binding appears to take place at least partially through the ganglioside’s pentasaccharide chain [102]. The reported dependence of anti-ganglioside antibodies on gluten intake would support such a mechanism of intermolecular help [96].

## 4. Synapsin I

The development of IgG and IgA antibody reactivity to gluten is a hallmark of celiac disease [11,14]. Whether anti-gluten antibodies can cross-react with autoantigens has been the subject of speculation and investigation for some time, especially in the context of extraintestinal manifestation of celiac disease. In a study by our group, we demonstrated that affinity-purified anti-gliadin antibodies from both immunized animals and celiac disease patients, particularly those with neurologic symptoms, can cross-react strongly with synapsin I (SYN1), a neuron-specific cytosolic phosphoprotein present in most nerve terminals [104]. The anti-gliadin antibodies bound to both isomers of SYN1, a and b, which have similar amino acid sequences. 

SYN1 is primarily associated with synaptic vesicle membranes at the cytoplasmic surface [105]. It is a major substrate for protein kinases and its state of phosphorylation can affect synaptic function [105]. The similarity in certain repeat amino acid sequences found in both gliadin proteins and SYN1, with high frequencies of proline and glutamine residues and the presence of PQP and PQQP motifs, is believed to contribute to the cross-reactivity between anti-gliadin antibodies and SYN1 [104]. While SYN1 is known to carry O-linked N-acetylglucosamine and fucosyl groups [106,107], the removal of these carbohydrates do not inhibit the binding of anti-gliadin antibodies to the protein, ruling out a major role for them as target epitopes.

Whether SYN1-cross-reactive anti-gliadin antibodies can exert a pathogenic effect in humans is unknown. Synapsins are multifunctional proteins, containing different domains with distinct activities [108]. In addition to binding synaptic vesicles and various cytoskeletal proteins [109,110,111], synapsins may have enzymatic functions as well [112,113]. Disruption of SYN1 activity via the use of anti-SYN1 antibodies to the aplysia sea slug homologue of synapsin has been shown to reduce post-tetanic potentiation, and to increase the rate and extent of synaptic depression [114]. However, sufficient data to clearly link anti-SYN1 antibodies to neurologic manifestations in the context of autoimmunity does not exist yet. The data from celiac disease patients demonstrate that only certain subsets of anti-gliadin antibodies cross-react with SYN1 [104]. Because of the large number and heterogeneous nature of gluten proteins and associated epitopes [115], the anti-gliadin immune response involves a diverse repertoire of antigenic determinants. Therefore, varying degrees of cross-reactivity to SYN1 would be expected in different individuals with an elevated immune response to gluten. Such differences in the degree of cross-reactivity may explain and reveal clues about the association of such antibodies with specific extraintestinal complications, including neurologic manifestations. Ultimately, the type and specificity of the immune response, local integrity of the blood–nerve and blood–brain barriers, and other pro-inflammatory factors are likely contribute to and influence the potential pathogenic role of SYN1-cross-reactive anti-gliadin antibodies [116]. It is worth noting that the pathogenic effect of anti-synapsin immune reactivity might not be limited to the nervous system, as the presence of low levels of SYN1 has been demonstrated in non-neuronal cells as well, including liver epithelial cells [117] and pancreatic β cells [118]. It is expected that these tissues would be more accessible to antibodies and T cells than the nervous system.

## 5. Other Autoantigens in Celiac Disease

A number of studies have reported on antibodies to cytoskeletal actin in the context of celiac disease [119,120,121]. Although these antibodies are not specific to celiac disease and have also been associated with chronic hepatitis [122,123], IgA anti-actin antibodies do appear to correlate with the degree of villous atrophy [121,124]. These findings suggest that anti-actin antibodies are linked with mucosal injury and may result from the release of actin from injured or dying cells, thus triggering an autoantibody response. In addition, their appearance in celiac disease is dependent on gluten intake [125]. It is not clear whether they are associated with or play any role in the extraintestinal manifestations of celiac disease. IgA autoantibodies to collagen types I, III, V, and VI have also been found in association with celiac disease. No specific clinical manifestation is reported to be associated with these antibodies yet, but the prevalence of connective tissue diseases in patients with celiac disease may be related to an anti-collagen immune reactivity. Antibody reactivity to single- and double-stranded DNA, ATP synthase β chain, cardiolipin, and enolase α has also been found in some celiac disease patients [120,126], but their clinical relevance remains to be assessed.

As celiac disease is associated with several other autoimmune disorders, including type 1 diabetes and autoimmune thyroiditis, autoantibodies specifically linked to these disorders can be found in patients with celiac disease [127]. A list of such antibodies and associated conditions is included in Table 1. The link between celiac disease and associated autoimmune disorders is believed to be primarily due to common genetic background, particularly in the HLA region of chromosome 6 [127,128,129,130]. Whether gluten intake can contribute to the development of these organ-specific autoantibodies is not entirely clear yet. However, there are reports showing that diabetes- and thyroid disease-related antibodies in children with celiac disease may disappear in response to the exclusion of gluten-containing foods from diet [131].

## 6. Autoantibodies in Non-Celiac Wheat Sensitivity

Some individuals experience a range of symptoms in response to the ingestion of gluten-containing foods, i.e., wheat, rye, and barley, without the characteristic serologic or histologic markers of celiac disease and wheat allergy [132,133,134,135]. The condition is variably referred to as non-celiac gluten sensitivity or non-celiac wheat sensitivity (NCWS). NCWS is associated with gastrointestinal symptoms, commonly including bloating, abdominal pain, and diarrhea, as well as extra-intestinal symptoms, among which fatigue, headache, anxiety, and cognitive difficulties are predominant [133]. Accurate figures for the prevalence of NCWS are not known, but current estimates put the number at similar to or greater than for celiac disease [136,137]. The identity of the exact component(s) of wheat and related cereals responsible for triggering the associated symptoms remains uncertain. While recent controlled trials have indicated a prominent role for gluten [134,138], non-gluten proteins and fermentable short chain carbohydrates have also been suggested by some studies to drive aberrant immune responses or to be associated with symptoms [139,140]. 

Recent research indicates that NCWS is associated with increased innate and adaptive systemic immune activation in response to microbial translocation [141]. Affected individuals also have elevated levels of intestinal fatty acid-binding protein that correlates with the markers of immune activation, suggesting a compromised intestinal epithelial barrier integrity [141]. By definition, NCWS patients do not have elevated levels of antibody to TG2, the celiac disease-specific autoantibody. Whether there is any autoimmune component to NCWS is not clear. However, one study has found that compared with irritable bowel syndrome (IBS) patients, an increased proportion of individuals with NCWS develops an autoimmune disease [142]. In addition, the study reported an increased frequency of anti-nuclear antibodies (ANA) in NCWS patients, as detected by indirect immunofluorescence using HEp-2 cells [142]. Anti-nuclear antibodies bind to proteins in the cell nucleus and are found to be elevated in some systemic autoimmune disorders, including systemic lupus erythematosus. The specific target antigen(s) and pathogenic relevance of these antibodies, or their association with specific extraintestinal manifestations, in the context of NCWS remains to be determined.

## 7. Conclusions

Celiac disease is a systemic autoimmune condition with intestinal and extraintestinal manifestations. Autoantibody reactivity against a number of autoantigens has been described in the context of the various manifestations of the disease. The clinical relevance and pathogenic role of such antibodies continue to be the subject of investigation and debate. Questions regarding the mechanisms by which such autoantibodies are generated and how they may access target tissues, such as the nervous system, also remain incompletely resolved. Identification of autoantibody biomarkers closely associated with specific extraintestinal manifestations of celiac disease can be particularly useful for the development of predictive and diagnostic tests, in addition to providing novel clues regarding disease mechanism and therapeutic approaches. Although a number of advances in the identification and understanding of autoantibody reactivity in celiac disease have been made in the past 20 years, further research and confirmatory studies with larger cohorts of patients and controls, as well as more in-depth preclinical mechanistic studies will be needed to clarify remaining questions.

## Figures and Tables

**Table 1:** Autoantibody reactivities associated with other autoimmune diseases found in patients with celiac disease.

Autoantibody Target	Associated Autoimmune Disease	Reference
Islet cells; Glutamic acid decarboxylase	Type 1 diabetes mellitus	[16,131,143]
Thyroperoxidase; Thyroglobulin, Thyroid stimulating hormone receptor; Thyroid microsomal antigen	Autoimmune thyroid disease	[16,131,144,145]
Liver-kidney microsomal antigen	Autoimmune liver disease	[16,146,147]
Double-stranded DNA; Nuclear antigen	Systemic lupus Erythematosus	[16,37,148]
Sjögren syndrome-related antigen A (Ro), Sjögren syndrome-related antigen B (La); Nuclear antigen	Sjögren syndrome	[16,149]
Cardiolipin; Phophatidylserine/prothrombin	Anti-phospholipid syndrome	[126,150,151,152,153]
